# The Utility of Recycled Rice Husk-Reinforced PVC Composite Profiles for Façade Cladding

**DOI:** 10.3390/ma15103418

**Published:** 2022-05-10

**Authors:** Ewa Sudoł, Ewelina Kozikowska, Emilia Choińska

**Affiliations:** 1 Construction Materials Engineering Department, Instytut Techniki Budowlanej, 00-611 Warsaw, Poland; e.kozikowska@itb.pl; 2 Faculty of Materials Science and Engineering, Warsaw University of Technology, 02-507 Warsaw, Poland; emilia.choinska@pw.edu.pl

**Keywords:** construction profiles, recycling, rice husk-reinforced PVC composite, performance properties, microstructure analysis, thermal and mechanical properties

## Abstract

The production process of construction profiles from natural fibre-reinforced polymer composites, as well as their assembly, generates considerable amounts of waste. The study analysed the possibility of utilising the said waste to produce profiles with the same intended use as products made from the primary material. The analysis involved the recycling of rice husk-reinforced PVC profiles. As a result of the applied post-processing, a composite of higher homogeneity and better filler dispersion than the product made of primary material was obtained. A slight improvement in thermal properties was observed. From the DSC test, Tg values of 78 °C and nearly 80 °C were obtained, while from the TGA test, mass loss values of 0.6% and 0.4% and the decomposition temperatures of 211 °C and 217 °C were noted for profiles of primary and secondary material, respectively. A softening temperature of 75 °C was obtained for primary material profiles, while nearly 77 °C was obtained for secondary. The more favourable mechanical properties of recycled profiles were also maintained. The higher value of flexural strength, flexural modulus, impact strength and hardness by 31%, 24%, 48% and 40% were obtained, respectively. After hydrothermal cycling, the same properties were higher for secondary material profiles by 35%, 20%, 68%, and 67%, respectively. The recorded level of performance properties of recycled products, better than those of primary material standard construction products’, allows us to conclude that profiles made of waste are useful for façade claddings.

## 1. Introduction

Many industries value the advantages of natural fibre polymer composites (NFPC). For years, they have played an important role in the automotive, aerospace and marine industries [1,2]. They are referred to as biocomposites because waste from wood and agricultural production [3,4], occasionally combined with raw material from recycled polymer products [5], is used in their manufacture. NFPCs are also gaining importance in building engineering [6]. Cellular and solid profiles are used for facade claddings, outdoor floors, or window and door joinery [7,8,9,10,11]. They are also used to construct walkways, promenades, street furniture and wet room furniture [12].

NFPCs dominate building engineering with polyvinyl chloride (PVC), high-density polyethene (PEHD) and polypropylene (PP) matrix [9,12]. The choice of a matrix is dictated by the application of the profile [13]. Wood fibres or fibres derived from husks, stems or leaves of mainly cultivated plants are usually used as reinforcement [14,15]. One of the most popular solutions in the construction industry is pulverised rice husk-reinforced PVC composite profiles [13,16,17]. They are usually produced by extrusion [18,19]. Profiles produced in this way have a smooth, slightly glossy surface. To give them a wood-like appearance, which visually increases the attractiveness of the products, and to improve the anti-slip properties, which are important for flooring applications [7], the usable surfaces of the profiles are surface treated [8]. This consists of grinding, brushing or milling.

Each of these processes generates waste. Grinding produces dust. Brushing and milling produce a coarser, chip-like waste fraction. Thanks to extractors installed on the production line, the collection of this waste is relatively simple. Waste is also generated in the next stage of the profiles’ life cycle, during their assembly. It then becomes necessary to cut them in order to achieve the correct fit of the component.

With the environment in mind, taking into account economic considerations as well as fulfilling obligations resulting from legal regulations, manufacturers of NFPC profiles strive to reduce the amount of recycled waste. Numerous actions are taken in order to manage them, including recycling aimed at manufacturing products with the same use as profiles made of the primary material [18,19,20,21].

The fitness for building engineering of NFPC profiles made from both primary and secondary raw materials is subject to verification according to the principles applicable to all construction materials. In line with the concept of sustainable development, fitness for construction is assessed on the basis of utility by identifying a set of key performance properties for a given application [22,23]. The assessment is carried out in the context of the product’s impact on the building’s compliance with the seven basic requirements [24], in accordance with Regulation (EU) No 305/2011 of the European Parliament and of the Council (CPR) [25]. With regard to façade cladding profiles, it is essential to determine the thermal and mechanical properties of the product, taking into account the influence of environmental factors.

Various research teams have addressed the recyclability issue of natural fibre-reinforced polymer composites. Shahi et al. [26] considered the recyclability of PEHD composite and wood flour profiles. After single processing, a reduction in filler fibre size accompanied by a reduction in interfacial adhesion was observed. What is more, a decrease in tensile strength and a slight increase in the flexural modulus was observed.

In contrast, Le Duigoua et al. [19] studied the recyclability of flax fibre poly(l-lactide) (PLLA) composites. They showed that post-processing using results in reducing fibre length while improving dispersion in the matrix. Already after the first injection cycle, a significant decrease in tensile strength, which deepened with the number of cycles, was observed. After six injection cycles there was a decrease. However, flexural modulus was quite stable.

Beg and Pickering [27] studied the recyclability of a composite with a PP matrix reinforced with wood fibres. They noticed the reduction in fibre length which worsened as the number of processing cycles increased. Decreases in tensile strength, flexural modulus, and impact strength were noticed.

On the other hand, Rosenstock Völtz et al. [18] conducted a study on the effect of post-processing on the properties of a composite with PP matrix and wood flour filler. It was found that post-processing resulted in a decrease in the fibre size of the filler, while increasing its dispersion in the matrix. What is more, an increase in interfacial adhesion in the microstructure analysis was observed. Repeating the process did not significantly affect the thermal properties, while the mechanical properties were less affected than in previous studies. The above results may be regarded as an indication that polypropylene composites with wood flour filler are recyclable without drastic changes in the properties.

When taking the above into account, it can be concluded that post-processing of natural fibre-reinforced polymer composites leads to a decrease in fibre length, which may result in a decrease in tensile strength, flexural strength and impact strength [18,26,27]. On the other hand, relatively small changes are observed in the flexural modulus, which is attributed to improved dispersion of fibres in the matrix [18,19]. The results of the works carried out so far confirmed the recyclability of NFPC products. However, it should be taken into account that the products made from recycled material may have different, possibly worse, performance properties. So far, the fitness of recycled profiles for building engineering has not been analysed. In this study, an attempt has been made to determine the level of performance properties of rice husk-reinforced PVC composite profiles, produced from waste from production and assembly, in the context of assessing their utility for façade cladding. A comparative study of standard construction profiles made from primary material was carried out. The composite microstructure, its thermal properties (DSC, TGA, softening temperature) and key mechanical properties for façade cladding (flexural strength, flexural modulus, impact strength and hardness) were analysed. The mechanical properties were determined for samples prepared in laboratory conditions as well as after hydrothermal cycling, taking into account the conditions of use of construction profiles and the adverse effects of atmospheric conditions on the mechanical properties of NFPC [28,29,30,31].

## 2. Materials and Methods

### 2.1. Profiles

For the purpose of the study, two series of profiles were produced:

- 20 profiles RO made of primary material in the form of commercial PVC composite granulate with a filler of pulverised rice husks;
- 20 profiles RZ made of secondary material, which is a composite of production waste:
  - In the form of dust with a diameter of about 100–200 μm and flat, irregularly shaped chips about 5–15 mm in size, from RO profiles’ surface treatment, constituting 50% by weight;
  - Shredded in the process of grinding assembly waste from RO profiles, in form of irregularly shaped particles about 3–7 mm in size, constituting 50% by weight.

The formulation of the primary material and the technology for obtaining the profiles are the manufacturer’s trade secret and has not been revealed. It was only found that in RO, the PVC matrix with a mineral filler in the form of chalk accounted for 40% by weight and the filler from rice husks, together with additives and modifiers, the remaining 60% by weight. The profiles were extruded in a plastic processing facility.

**Figure 1:**
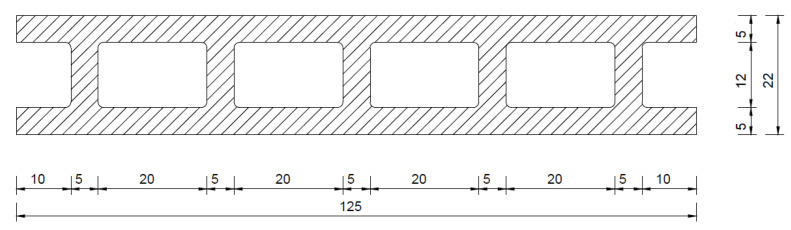
Shape of the profiles used in the tests.

The profiles of both series had the same shape. Their overall width was 125 mm, height 22 mm, face wall thickness 5 mm and cell width 20 mm (Figure 1). The profiles had two usable surfaces, which were subjected to a standard grinding treatment.

**Figure 2:**
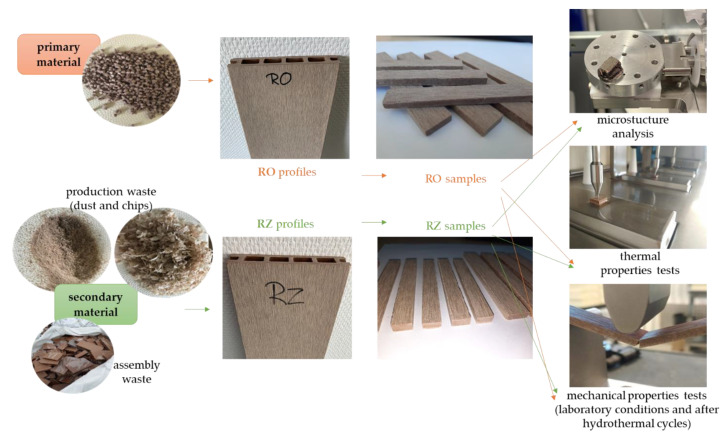
Experimental flowchart with photos.

Samples with a width less than 20 mm were cut from the usable surface of the profiles, from the central part of the cells, between the vertical ribs parallel to the cells. Samples with a width of more than 20 mm were cut from the same surfaces after removing the ribs. Figure 2 shows the research flowchart with some photos of the experimental steps.

### 2.2. SEM Analysis

The microstructure of composite profiles was examined with Sigma 500 VP cold-field emission scanning electron microscope (Carl Zeiss Microscopy GmbH, Köln, Germany), The tests were carried out at the accelerating voltage of 5 or 10 KeV inductive electron beam, using an SE detector on samples coated with a gold film. Observations were carried out at 200 and 5000× magnification. The microstructure of the composites was observed on brittle fractures obtained under laboratory conditions (the temperature 23 ± 2 °C). Tests were performed on profile samples obtained from the primary material (RO) and secondary material (RZ).

### 2.3. Thermal Properties

Thermal properties were investigated on samples obtained from profiles made of primary material (RO) and secondary material (RZ). The differential scanning calorimetry (DSC), thermogravimetry (TGA) and softening temperature were performed.

DSC analyses were performed using the Q2000 apparatus (TA Instruments, New Castle, DE, USA). Flat pieces of extruded profiles weighing about 12–15 mg were crimped in aluminium pans. The measurements were performed in the temperature range 0–250 °C at a nitrogen flow of 50 mL/min. The constant heating rate of 10°/min was applied. Three samples of each material were tested. The glass transition temperature (Tg) was determined as a half-height of the transition.

TGA was carried out using the Q5000IR device (TA instruments, New Castle, DE, USA). Samples, three for each material, were heated up to 1000 °C at a nitrogen flow of 25 mL/min. The constant heating rate of 10°/min was applied.

The softening temperature was determined using the Vicat method (VST), according to ISO 306 [32]. The temperature at which a steel needle, applied to the ground face of the profile, penetrated to a depth of 1 mm under a load of 50 N was determined. Silicone oil was used as the heating medium, the temperature of which, after immersing the samples in the bath, increased at a rate of 50 K/h.

### 2.4. Mechanical Properties

Mechanical properties were tested on samples obtained from profiles made from the primary material (RO) and the secondary material (RZ). The flexural strength, flexural modulus, impact strength and hardness were tested. The research was carried out with the test methods previously used by Sudoł et al. [8]. All properties were determined for samples prepared in laboratory conditions and after hydrothermal cycling according to EN 15534-1 [33] for 56 days:

- 600 h immersion in water at the temperature of 23 ± 2 °C;
- Three cycles, each consisting of:
  - 72 h immersion in water at the temperature 23 ± 2 °C;
  - 24 h freezing at the temperature −20 ± 2 °C;
  - 72 h drying at the temperature 70 ± 2 °C.

The flexural strength was tested according to ISO 178 [34], using a class 1 strength testing machine (Instron, Darmstadt, Germany). Three-point bending was performed, using samples sized 15 × 100 × 5 mm. Supports with a 5 mm radius were used, spaced every 80 mm, corresponding to 16-times sample’s thickness and a 5 mm radius pressing element placed in the middle of the span. The samples were freely supported. The load was applied to the ground surface at a constant rate of 5 mm/min until destruction. Flexural strength *σ_f_* was calculated according to (1) and expressed in Mpa [8]. Twelve samples were tested in each series, giving a total of forty-eight samples tested in the study.

(1) $$\sigma_{f}=\frac{3FL}{2bh^{2}}$$

where the following are defined: *F*—maximum force, in N; *L*—support spacing, in mm; *b*—sample’s width, in mm; *h*—sample’s thickness, in mm.

The flexural modulus was also tested according to ISO 178 [34], using a class 1 strength testing machine (Instron, Darmstadt, Germany), in conditions identical to flexural strength tests. A load-deflection curve was recorded during bending in a linearly elastic range, including the force and deflection values corresponding to strain ε*_f_*_1_ = 0.0005 and ε*_f_*_2_ = 0.0025. The *f*_1_ and *f*_2_ deflection values were calculated according to (2).

(2) $$f_{1}=\frac{\varepsilon_{f1}L^{2}}{6h};\;f_{2}=\frac{\varepsilon_{f2}L^{2}}{6h}$$

where the following are defined: *L*—spacing of supports, in mm; *h*—sample’s thickness, in mm.

The force values recorded when ε*_f_*_1_ and ε*_f_*_1_ strain occurred were used for determining the values of σ*_f_*_1_ and σ*_f_*_2_ normal stress. The *E_f_* modulus was calculated according to (3) and expressed in Mpa [8]. Twelve samples were tested in each series, giving a total of forty-eight samples tested in the study.

(3) $$E_{f}=\frac{\sigma_{f2}-\sigma_{f1}}{\varepsilon_{f2}\;-{\;\varepsilon }_{f1}}$$

where the following are defined: σ*_f_*_1_, σ*_f_*_2_—maximum normal stress corresponding to *f*_1_ and *f*_2_ stress determined according to (2).

The impact test was carried out with the Charpy impact pendulum (ZwickRoell, Ulm, Germany) according to ISO 179-1 [35]. The samples used in the test had no notch, were sized 10 × 80 × 5 mm. The sample was freely resting on supports spaced at 62 mm and then hit with a 2J impact pendulum. The load was exerted on the ground surface. Impact strength *a_cU_* was calculated according to (4) and expressed in kJ/m^2^ [8]. Twelve samples were tested in each series, giving a total of forty-eight samples tested in the study.

(4) $$a_{cU}=\frac{E_{c}}{h\cdot b}\cdot 10^{3}$$

where the following are defined: *E_c_*—energy absorbed by breaking the test specimen, in J; *h*—sample’s thickness in mm; *b*—sample’s width, in mm.

The hardness test was performed using the Brinell method according to EN 1534 [36]. Samples with dimensions of 50 × 50 × 5 mm were used. The test consists of pressing a steel ball into the ground surface of the profile in order to indent it permanently. A 10 mm diameter ball was used, and a load of 1840 N was maintained for 25 ± 5 s. Then, 15 s after removal of the load, the diameter of the imprint left by the ball was measured using an optical microscope. The hardness was calculated according to (5) and expressed in MPa. Ten samples were tested in each series. A total of forty samples were tested in the experiment.

(5) $$HB=0.102\frac{2F}{\pi \;D\;(D-\sqrt{D^{2}-d^{2})}}$$

where the following are defined: *F*—force, inN, *D*—ball diameter, in mm, *d*—imprint diameter, in mm.

## 3. Results and Discussion

### 3.1. Microstructure Analysis

**Figure 3:**
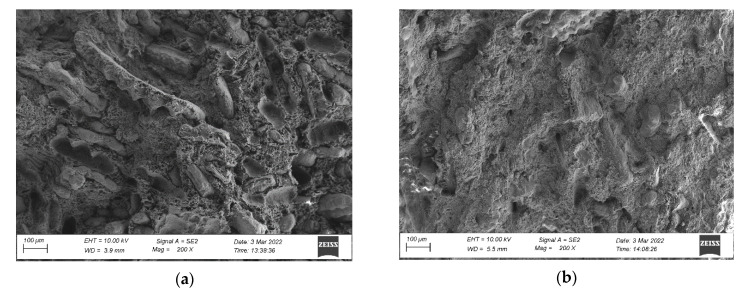

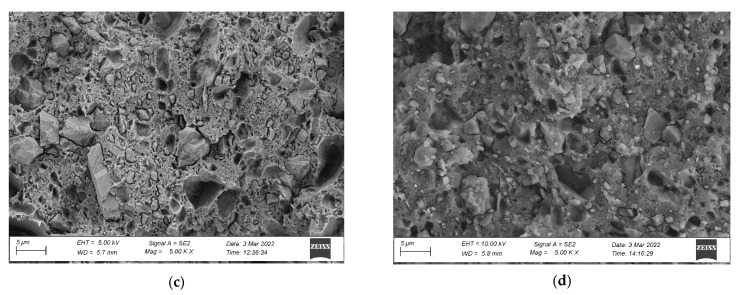
Microstructure of fracture surface, at 200× magnification, obtained for (**a**) RO primary material, (**b**) RZ secondary material and at 5000× magnification, obtained for (**c**) RO primary material, (**d**) RZ secondary material.

The microstructure of the composites was performed on brittle fractures to evaluate the degree of dispersion of pulverised rice husks and mineral filler (chalk) in the PVC matrix. Pulverised rice husks can be seen on microscopic images in the form of fibres and lamellae, which are larger fragments of uncrushed rice husks (Figure 3a,b). Analysis of the microstructure indicates that the applied post-processing improved the homogeneity of the composite, which is attributed to improved interactions at the polymer-filler interface [14,37]. The structure of the RO composite was much less homogeneous than that recycled composite RZ. In the RO composite numerous voids of extracted filler particles were observed (Figure 3a), indicating its low adhesion to the polymer matrix [14]. The RZ composite showed no such trend. In RZ composite, the mineral filler was dispersed more uniformly than in RO, and consequently its agglomerates were significantly smaller and better wetted by the polymer (Figure 3c,d).

High homogeneity at the interface of composites leads to improvements in both their mechanical performance and water resistance [38]. The physico-mechanical and thermal properties of polymer composites depend significantly on the fineness of the filler particles, its shape and its uniform dispersion in the polymer [18,39]. Obtaining a composite with a uniform particle distribution is difficult due to its small size and tendency to form agglomerates. At the same time, filler agglomeration causes insufficient wetting of the ceramic by the polymer, which translates into a decrease in functional properties [14,39]. Proper fibre dispersion in the matrix develops good interfacial adhesion and thus reduces voids, ensuring that the fibres are fully surrounded by the polymer matrix [5,14].

### 3.2. Thermal Properties

**Figure 4:**
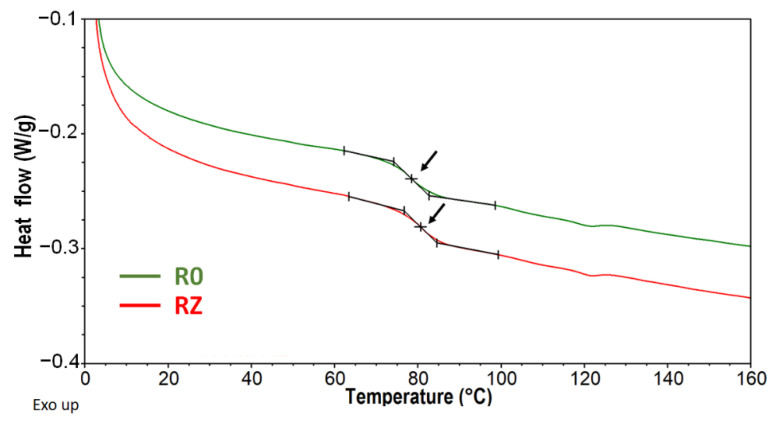
DSC curves obtained for RO and RZ composite materials (arrows point the glass transition).

**Figure 5:**
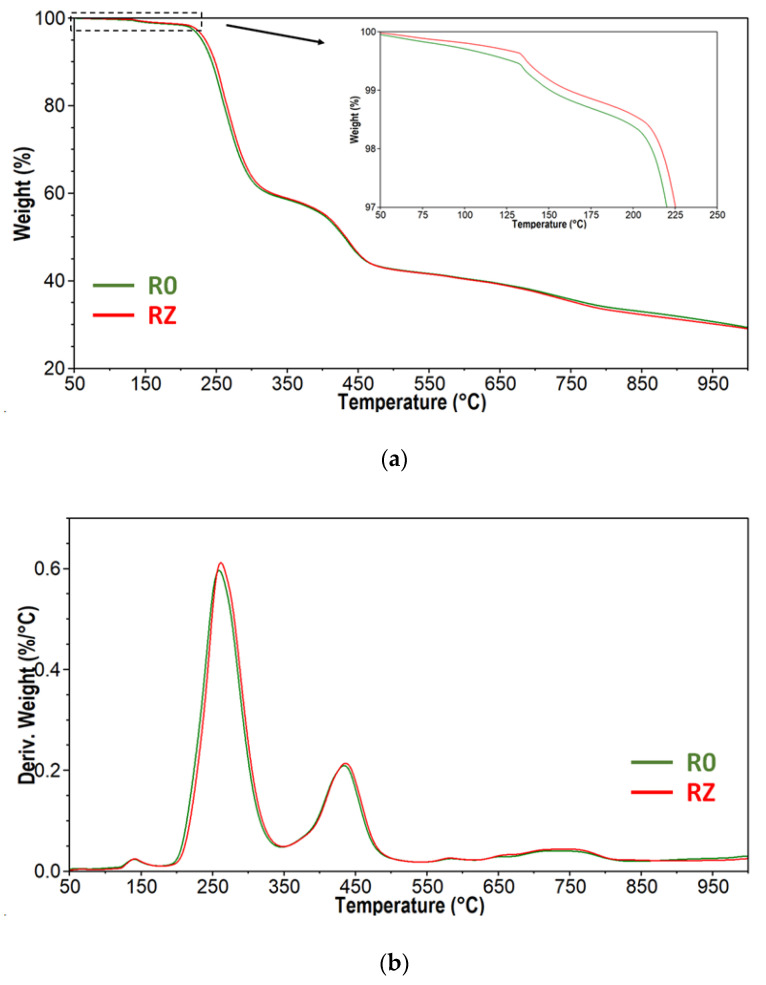
TGA (**a**) and DTGA (**b**) curves obtained for RO and RZ composite materials.

It is known that the thermal properties of recycled materials can be affected by the presence of degraded polymers. These low molecular weight fractions can act as plasticisers, resulting in lower glass transition temperature values [40,41]. In the present study, both materials, made from primary granulate and made from the waste, showed similar behaviour during the DSC test (Figure 4). The determined Tg values were 78.3 ± 0.4 °C and 79.9 ± 0.6 °C for RO and RZ, respectively. It suggests that the polymer matrix was not degraded during the grinding, brushing, milling and secondary extrusion processes. Moreover, the slight increase in glass transition temperature was observed, which is probably an effect of removing residual water or other thermally unstable components from rise husks [42,43]. This effect was observed also during TGA measurements (Figure 5a). Both materials have shown the same degradation profile; however, the samples made of primary granulate revealed slightly bigger mass loss at the beginning of the pyrolysis process. At 130 °C, it was 0.6 ± 0.04% and 0.4 ± 0.02% for RO and RZ, respectively. This difference is small; however, it can explain the above-mentioned increase in Tg. Moreover, the samples made of production wastes were a bit more thermally stable. The decomposition temperature, defined as the temperature of 2% mass loss (T_d2%_), was 211.1 ± 1 °C and 216.7 ± 1.3 °C for RO and RZ, respectively. It seems that due to the waste-generating processes, but probably mainly due to the secondary extrusion process (thermal process), the volatile residues present in components of composite material were evaporated. Nevertheless, it does not have an effect on the character of pyrolysis. For both materials, the decomposition process is composed of few steps, which is clearly presented on the DTGA curves (Figure 5b). The first one is observed up to about 190 °C and is an effect of free water and water bonded by hydrogen bonds evaporation [44,45]. The second, most intensive one, which proceeds between 190 °C and 350 °C, can be assigned to the overlapped processes of the hemicellulose from rice husks degradation and dehydrochlorination of PVC matrix and creation of polyene structure [46]. The next stage is observed between 350 °C and 520 °C. In this temperature range, the degradation of cellulose and lignin appears [44,45] and decomposition of polyene structure [46]. Above 520 °C, an additional wide and complex step of pyrolysis is observed. Any organic additives and residues which were not degraded at lower temperatures are decomposed at this stage, and char is formed.

Differences were observed for the softening temperature. A VST of 75 °C was obtained for RO profiles, while a VST of nearly 77 °C was obtained for RZ profiles. Both of these values are similar to the values obtained for façade profiles [47].

Overall, it can be said that the improvement in thermal properties, although slight, was observed. Such favourable results have not been recorded in previous research. The most important aspect is that usage of the recycled composite allowed us to obtain the profiles with no worse thermal properties than primary material profiles, standardly used in façade cladding.

### 3.3. Mechanical Properties

**Figure 6:**
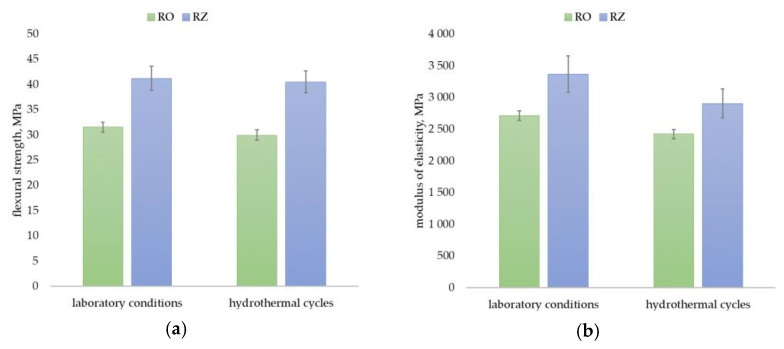
Test results for (**a**) flexural strength, (**b**) flexural modulus. Error bars represent standard deviation.

The flexural properties are one of the key performance properties for façade cladding profiles that are normally installed as point-supported sections on the grid [9,47]. Both their flexural strength and their flexural modulus, which express the stiffness of the material and thus show the profiles’ susceptibility to deformation under service loads, are important. Analysis of the test results obtained shows that the flexural strength of the profiles made from primary material was 32 MPa in the initial state and 30 MPa after hydrothermal cycling, while that made from the secondary material was 41 MPa and 40 MPa, respectively (Figure 6a). These values for those properties are lower than those obtained by Vercher et al. [13] for construction profiles made of composites with PE and PVC matrix both with rice husks, for which 71 MPa and 67 MPa were recorded, respectively. Significantly higher values for construction profiles made of polyester matrix composite reinforced with sycamore, sisal or bamboo fibres were also obtained by Prasad and Rao [48]. Their flexural strengths ranged from 100 MPa to 134 MPa. The tested material has a lower value of flexural strength than that reported by Sudoł et al. [8] for profiles made of PVC and wood flour composite, for which the value is 67 MPa. The values of flexural strength for RO and RZ profiles, on the other hand, are higher than that established in a study by Stark [31] conducted for composites with a matrix of recycled high-density polyethylene and a filler of rice husks. At a filler content of 50%, a flexural strength of 25 MPa was obtained. The flexural modulus of the RO profiles was 2710 MPa in its initial state and 2420 MPa after hydrothermal cycling, while that of the RZ profiles was 3360 MPa and 2900 MPa, respectively (Figure 6b), which can be considered a favourable result in light of the previous studies. The value for construction profiles made of composite with HDPE matrix and wood flour recorded by Stark [49] is 2530 MPa, while by Pilarski et al. [50] it is 3600 MPa. On the other hand, for profiles with PEDH matrix reinforced with sisal and bamboo fibres, Prasad and Rao [48] obtained 2500 MPa and 3700 MPa, respectively. Stark [31], on the other hand, reported 2600 MPa and 2800 MPa for composites with recycled PEHD matrix and rice husks filler at 50% and 70% filler content, respectively.

**Figure 7:**
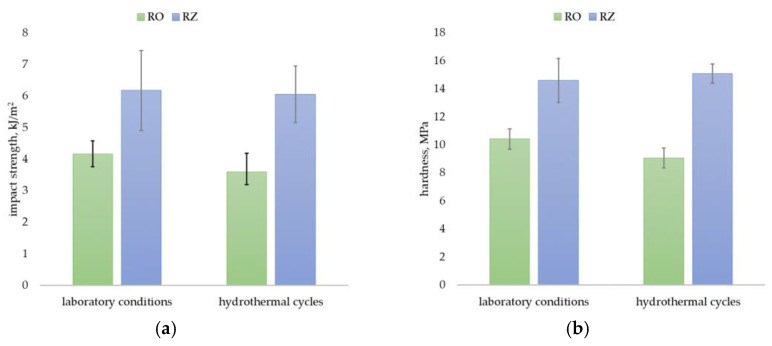
Test results for (**a**) Charpy impact strength, (**b**) Brinell hardness. Error bars represent standard deviation.

Because of the high risk of construction profiles’ exposure to dynamic loads throughout their entire life, a stable impact strength value expressing the material’s susceptibility to fracture can be considered one of the key performance properties [8,37]. The RO profiles tested in this study have an impact strength of 4.2 kJ/m^2^ after preparation in a laboratory and 3.6 kJ/m^2^ after hydrothermal cycling, and the RZ profiles have 6.2 kJ/m^2^ and 6.0 kJ/m^2^, respectively (Figure 7a). All samples fractured completely at the point of application of the hammer drill, i.e., they showed brittleness [35]. The impact strength values determined in this study are similar to those obtained by Turku et al. [38], who studied construction profiles made of wood plastic composites with PEHD waste. These products had impact strength ranging from 2.9 kJ/m^2^ to 8.5 kJ/m^2^, depending on the proportion of PEHD. However, the results of the work by Sudoł et al. [8] indicate that selected construction profiles made of PVC composite with wood flour filler have much higher value of impact strength, exceeding 20 kJ/m^2^.

The hardness of RO profiles was 10.4 MPa after preparation under laboratory conditions and 9.1 MPa after hydrothermal cycling, while that of RZ profiles was 14.6 MPa and 15.1 MPa, respectively (Figure 7b). These values are far superior to those reported by Turku et al. [38] for profiles made of wood plastic composites with PEHD waste, for which 3.7 MPa to 5.1 MPa were obtained.

A comparison of the properties of products made from secondary material (RZ) and primary material (RO) indicates that the post-processing used in this study had a beneficial effect on the mechanical properties. Profiles RZ showed a 31% higher flexural strength, 24% higher flexural modulus, 48% higher impact strength and 40% higher hardness compared to RO. After hydrothermal cycling, those properties for RZ profiles were higher than RO by 35%, 20%, 68%, and 67%, respectively. Such significant increases in values have not been observed in previous works. For a PP matrix composite reinforced with wood fibres, Beg and Pickering [27] reported a 30% decrease in flexural strength, 15% decrease in flexural modulus and 50% decrease in impact strength after post-processing. Rosenstock Völtz et al. [18] reported a 20% decrease in tensile strength with essentially unchanged value of flexural modulus for the composite with PP matrix and wood flour filler. It should be noted that in the cited works, the composite underwent eight and nine processing cycles, while in the present work the composite was post-processed only once. Shahi et al. [26] also observed an increase in flexural modulus after single processing.

The mechanical properties of polymer composites depend significantly on the filler particles’ fineness, shape, and degree of dispersion in the polymer matrix [12,15]. The reported increase in mechanical properties due to the applied post-processing indicates that this process resulted in obtaining a composite with a more homogeneous structure, which was also observed in earlier works [18,26,27]. The above is confirmed by the microstructure images. The secondary material RZ showed smaller filler agglomerates (Figure 3b,d), better matrix surrounding, and reduced voids compared to primary material RO (Figure 3a,c). However, no significant reduction in fibre size, which has been attributed to adverse effects on flexural strength and impact strength, was observed [26,27].

The hydrothermal cycles carried out resulted in a decrease in mechanical properties. A decrease in flexural strength of 5% for the RO profiles and less than 2% for the RZ profiles was recorded. The flexural modulus decreased by nearly 11% for RO and 14% for RZ. In the case of impact strength, the decrease was 14% for RO and 2% for RZ. Hardness for RO decreased by 13%, and for RZ, there was an unexpected increase of 3%. It should be noted that the applied hydrothermal cycles included, among others, prolonged exposure to water, which can have a very adverse effect on the functional properties of polymer composites with plant particle filler [28,29,30,31]. The hydrophilic nature of plant fillers causes them to swell in a water environment leading to the formation of cracks in the hydrophobic polymer matrix [51]. The cooperation between cellulose fibres and polymer also deteriorates. Insufficient adhesion at the interface leads to a consequent decrease in mechanical performance [52]. Friedrich’s [9] analysis of an extensive dataset of changes in the flexural properties of natural fibre-reinforced polymer composites under the influence of environmental factors led to the conclusion that hydrothermal cycling using a procedure similar to that used in this work, can cause a decrease in flexural strength and flexural modulus of up to 8% and 25%, respectively. Therefore, it can be concluded that the products tested in this study, made of both primary material (RO) and secondary material (RZ), obtained a higher than standard resistance to hydrothermal cycling. It should also be emphasised that after hydrothermal cycling, the profiles made of secondary material had 35% higher flexural strength, 20% higher flexural modulus, 68% higher impact strength and 67% higher hardness than those made of primary material. Such good results have not been recorded in previous studies. The above may be due to the observed better surrounding of the filler with the matrix in secondary composite than primary composite. It can be assumed that the hydrophobic filler particles in the recycled material are more protected by the polymer matrix against moisture-induced swelling than in primary composite.

## 4. Conclusions

The data analysis obtained in this study indicates that the waste from the production and assembly of rice husk-reinforced PVC composite products can be used to manufacture façade claddings. As a result of the applied post-processing, profiles of higher homogeneity than the primary material products were obtained. In the samples of primary material numerous voids of extracted filler particles were observed. The samples of secondary material showed no such trend. In recycled composite, fillers were dispersed more uniformly, and consequently its agglomerates were significantly smaller and better wetted by the polymer. A slight improvement in thermal properties was observed. From the DSC test, a Tg of 78 °C was obtained for profiles of primary material and nearly 80 °C for secondary material. During TGA measurements, both materials have shown the same degradation profile; however, the profiles made of primary material revealed slightly bigger mass loss at the beginning of the pyrolysis process. At 130 °C, it was 0.6% and 0.4% for products of primary and secondary material, respectively. Moreover, the profiles made of production wastes were a bit more thermally stable. The decomposition temperature was 211 °C and 217 °C for profiles of primary and secondary material, respectively. Softening temperature of 75 °C was obtained for profiles of primary material, while it was nearly 77 °C for secondary material. More favourable mechanical properties of the recycled composite were also observed. Profiles of secondary material showed a 31% higher flexural strength, 24% higher flexural modulus, 48% higher impact strength and 40% higher hardness compared to profiles of primary material. It should be noted that such significant increases in values have not been observed in previous works. It must also be emphasised that the favourable mechanical properties of the secondary material were obtained not only in the initial state, but also after exposure to hydrothermal cycling, which are considered to be particularly unfavourable for polymer composites reinforced with hydrophilic plant fibres. After hydrothermal cycling, those properties were higher for profiles of secondary material by 35%, 20%, 68%, and 67%, respectively. The recorded level of performance properties of recycled products, better than the properties of standard construction products made of primary material, allows one to conclude that profiles made of waste are useful in construction to the same extent as profiles made of original material. The recycled profiles tested at work were introduced to the market.

The work does not exhaust the subject of the utility of recycled profiles of waste products from natural fibre-reinforced polymer composites. When considering the dynamic nature of these materials and their growing importance in building engineering, further study is planned. Further research will consider other NFPC products and post-processing methods.

## Data Availability

The data presented in this study are available on request from the corresponding author.
